# Imaging of Ultra-Weak Photon Emission in a Rheumatoid Arthritis Mouse Model

**DOI:** 10.1371/journal.pone.0084579

**Published:** 2013-12-30

**Authors:** Eduard van Wijk, Masaki Kobayashi, Roeland van Wijk, Jan van der Greef

**Affiliations:** 1 Sino-Dutch Centre for Preventive and Personalized Medicine/Centre for Photonics of Living Systems, Leiden University, Leiden, The Netherlands; 2 Department of Electronics and Intelligent Systems, Tohoku Institute of Technology, Sendai, Japan; 3 Division of Analytical Biosciences, Netherlands Metabolomics Centre, LACDR, Leiden University, The Netherlands; 4 TNO Netherlands Organization for Applied Scientific Research, Zeist, The Netherlands; 5 Meluna Research, Geldermalsen, The Netherlands; 6 Samueli Institute, Alexandria, Virginia, United States of America; MGH, MMS, United States of America

## Abstract

Ultra-weak photon emission (UPE) of a living system received scientific attention because of its potential for monitoring increased levels of reactive oxygen species (ROS) in the pathogenesis of rheumatoid arthritis (RA). In this study, a highly sensitive cryogenic charge-coupled device (CCD) camera was used to monitor in a RA mouse model the photon emission both without and with luminol. For that purpose, arthritis was induced in mice utilizing a repeated co-administration of type II collagen with lipopolysaccharide. Quantitative imaging of ultra-weak photon emission of the front and back paws of the animals was initiated 70 days after the first injection. All of the animals were measured once without luminol and once again immediately after luminol injection. Data illustrated a higher UPE intensity after initiating arthritis by CII-injection of the animals. The increase in UPE intensity was measured with and without using luminol indicating that this imaging technology may be useful for the future study of human RA.

## Introduction

Convincing evidence supports a role for oxidative stress and the subsequent production of reactive oxygen species (ROS) in the pathogenesis of many chronic diseases. The importance of ROS has stimulated the development of techniques for their estimation and evaluation of therapeutic interventions. In particular, the techniques that can be applied both non-invasively and locally estimating radiation energy vis-à-vis documentation of photon emission in the UV, visible and near IR ranges. Since the 1980’s, many experiments have revealed that weak photon emission could originate from natural biological reactions of free radicals and their derivatives, and also from simple cessation of electronically excited states. As examples may be listed the mitochondrial respiration chain, lipid peroxidation, peroxisomal reactions, oxidation of tyrosine and tryptophan residues in proteins, etc. [1]–[6]. One of the major sources of weak biological photon emission is mitochondrial oxidative metabolism and lipid peroxidation. It is due to the excited electrons of singlet oxygen ^1^O_2_ and carbonyl species R = O^*^. When an excited carbonyl or singlet oxygen is released to the ground state, it can emit its energy as a photon in the visible range. Photon emission from dimole emission of singlet oxygen (^1^O_2_+^1^O_2_ → 2 ^3^O_2_+ *hν*) and carbonyl species (R = O^*^ → R = O+*hν*) range in the order of 634–703 nm and 450–550 nm, respectively. The origin of weak radiation was also frequently discussed from the point of view that, usually, only primary emission emanating from the surface would be measured. Emission occurring in deeper layers may be absorbed and become part of the transmission of excited states, both dark and light, the latter resulting in secondary radiation from other sources [7], [8].

Early studies have already indicated that this technique (utilizing photomultipliers and photon emission imaging equipment) could serve as a useful biological marker in order to detect physiological malfunctions in tumor development. In cell studies, experimental data pointed to higher primary and secondary emissions of tumor cells compared to normal parental cells [9]–[13]. The data have been confirmed utilizing normal and tumor tissue [14]. The use of two-dimensional imaging plus photon counting of ultra-weak photon emission (UPE) from a transplanted bladder cancer into the feet of nude mice was reported in 1995 [15]. During the early log phase of cancer cell growth (prior to necrosis, hemorrhage, and leukocyte infiltration) increased photon emission was observed in the implanted tumor region indicating emission from the actively proliferating cancer. Other data confirmed the increased photon emission of tumors [16]. Utilizing a highly sensitive, ultra-low-noise charge-coupled device (CCD) camera system, these authors also recorded UPE from mice at the site that ovarian cancer cells were transplanted.

Increased oxidative stress also plays a significant role in the pathogenesis of Rheumatoid Arthritis (RA) [17]–[20]. The same biological marker has been seen in both rheumatoid joint synovial fluid and tissue where oxidative products are elevated and antioxidants are reduced [21]–[23]. Since weak photon emission is correlated with oxidation processes, it means that rheumatoid arthritis might also be studied by photon emission imaging methods. It was demonstrated that it is feasible to image increased ROS levels non-invasively in a RA mouse model using luminol (intravenously) which, then, acts as a documenter of ROS [24]. Luminol (3-aminophthalic hydrazide) is a small molecule photon donor which is activated by ROS resulting in more effective photon emission [25]. However, the effective use of luminol in human research subjects is highly unlikely. Therefore, a non-invasive method without the use of an enhancer substance for the early detection of arthritis development by monitoring ultra-weak photon emission would undoubtedly be very helpful for both basic arthritis research and its clinical management of human patients. The development of such a method would be a challenging multistep process. Therefore, the aim of such a study would be to image UPE both without and with the enhancer luminol utilizing an experimental mouse model for rheumatoid arthritis.

## Materials and Methods

### Ethics Statement

This study was carried out in strict accordance with the recommendations in the Guide for the Care and Use of Laboratory Animals of the National Institutes of Health.The experiments were performed with the approval of the Tohoku Institute of Technology Research Ethics Committee, Sendai, Japan (approval date 18 January 2009).

### Animals

DBA/1J mice are widely used as an animal model to investigate rheumatoid arthritis [26], [27]. In this model, immunization with collagen (Type II) will provoke severe polyarthritis by the induced autoimmune response. Twenty (10 control; 10 experimental) male DBA/1J mice, 6–7 weeks of age, were utilized in this study. The mice were obtained from Charles River, Yokohama, Japan. They were maintained in a temperature and light controlled environment with free access to standard rodent chow and water.

### Induction of Arthritis by Co-administration of Type II Collagen (CII) with Lipopolysaccharide (LPS)

In experimental animals, RA was induced by co-administration of type II collagen (CII) with lipopolysaccharide (LPS). One hundred micrograms of CII extracted from bovine nasal cartilage (Funakoshi Co., Tokyo, Japan) was dissolved in 100 µl of 0.005 M acetic acid and injected intraperitoneally (i.p.) into mice (day 0). Thereafter, the CII injection was repeated i.p. on days 14, 28, 42 and 56. In the control mice, 100 µl of 0.005 M acetic acid alone was administered i.p. on the same days.

In the experimental mice, 5 µg of LPS from E. coli 011:B4 (Chondrex, Redmond, USA) dissolved in 100 µl phosphate buffered saline (PBS) was also given i.p. immediately after each injection of CII. In the control animals, 100 µl PBS was similarly administered as a control. This protocol for arthritis induction is extensively described [28].

### CCD Camera System

**Figure 1:**
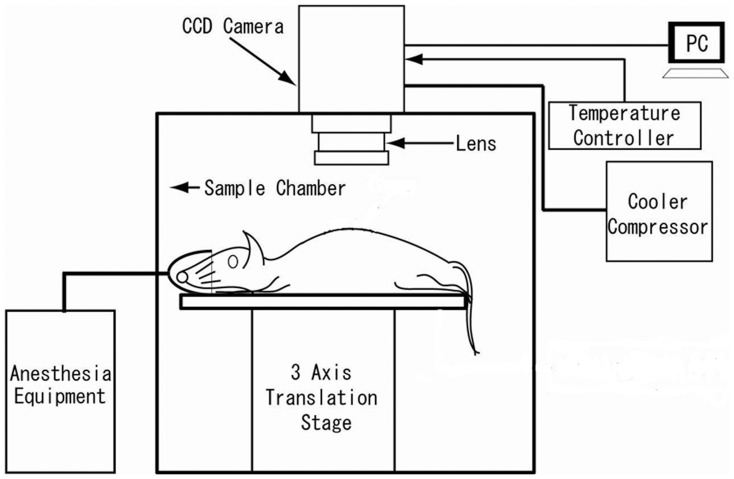
Schematic representation of the experimental set-up.

Spectral Instruments 600 series CCD camera system (Spectral Instruments, Inc., AZ, USA) was used. It has a mounted CCD42-40 (e2v technologies Ltd., Essex, UK), which was back-illuminated with a full frame operation CCD with 2048×2048 pixel resolution and 13.5×13.5 µm pixel size. The camera system is equipped with a cooling head to maintain the CCD at –120°C utilizing a closed-cycle mechanical cryogenic unit. Under these conditions, quantum efficiency is 75% at the peak wavelength. Dark current is 0.65 electron/pixel/h and readout noise in the slow scanning mode is less than 4.5 electron rms. The CCD camera head had a specially designed lens system, which was designed to maximize the light collection efficiency (numerical aperture of the lens system on the detector side is 0.5 and the number of lenses was restricted to 7 pieces). Magnification of the lens system is ∼1/2. In this experiment, the CCD was operated in the 16×16 binning mode. The actual pixel number was 128×128. Taking into account the detection limit, which was determined by dark current and readout noise of the CCD as well as light collection efficiency, the minimum detectable number of photons on each pixel was estimated ca 47 photon/s/cm^2^ on the surface of the subject under the measurement condition. The CCD camera was mounted on the top of a dark chamber (Figure 1). The dark chamber was free from any phosphorescent or synthetic colour. The temperature in the recording chamber was maintained at 20±1.0°C.

### Determination of Ultra-weak Photon Emission (UPE)

Ultra-weak photon emission measurement of the front and back paws of the animals started 70 days after the first injection [28]. All of the animals were measured once without luminol and once immediately after luminol injection. Prior to the recording the animals were anesthetized with isoflurane with the purpose of keeping animals in exactly the same position during the measurement. A position image was made under weak light illumination before the actual imaging of UPE. It was checked that this weak illumination has no influence on the actual imaging of UPE. Subsequently, the front area of the animals (focusing on the front paws) was recorded, immediately followed by the recording of the back part (focusing on the back paws). The time for each UPE recording was 15 minutes.

Two days after UPE recording without luminol, the animals were again recorded for luminol-enhanced luminescence. The animals were anesthetized and then injected intravenously with 5 mg luminol (3-aminophtalic hydrazide) dissolved in 100 µl PBS. Recording started immediately thereafter using the above recording protocol. No adverse effects of the luminol were observed in mice injected at doses of 250 mg/kg [29].

### Data Analysis

**Figure 2:**
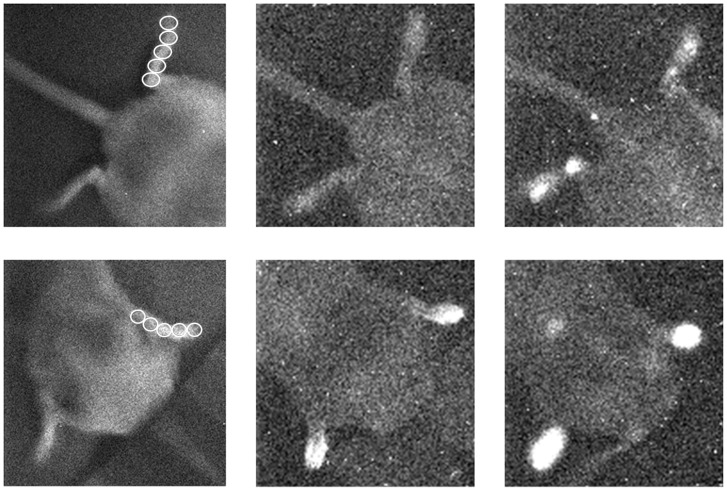
Image of left and right hind paws (upper panel) and front paws (lower panel) of experimental mouse 7. In both rows, the left image is a position image, recorded under weak light illumination before the actual imaging of UPE. Middle images represent UPE before luminol injection. Right images represent UPE immediately after luminol injection.

Each paw’s image was divided into 5 regions of interests (ROI): ROI 1 through ROI 5 with ROI 1 closest to the tip of the paw and ROI 5 closest to the joint of the paw. The other ROI’s were chosen in such a way that they equally covered the paws between tip and joint (as marked in the left panel of Figure 2 A and B). The surface for the ROI’s chosen for the paws was a circle covering 21 pixels (in the case of the front paws) and 32 pixels (in the case of the hind paws). The off-set level of read-out amplifier is subtracted from the observed values. In the way described above, photon emission from each paw can be described by a mean intensity (counts/15 min/pixel) and standard deviation.

## Results

### Visual Inspection of CCD Images

CCD imaging started 70 days after RA induction. Visual inspection of these images documented that in both control and CII-injected mice, the front paws emitted a higher emission than the hind paws. In both hind and front paws, the emission after luminol injection was higher than before the luminol injection. Figure 2 is a representative example of UPE images registered from the hind and front paws of a single animal. They suggested a variation in the intensity *between* and *within* the 4 paws of an animal. This variance in intensity was higher in the experimental animals as compared to the control animals. Such variance within an animal increased after luminol injection. These observations were quantified according to the protocol described in the data analysis section.

### Quantitative Description of CCD Images

Mean UPE intensity of both experimental (CII injected) and control group were compared before and after injection of luminol. Before luminol injection, the average intensities of front and hind paws of individual mice were in the range of 26–80 and 23–61, respectively. In the visual inspection of the images of both control and CII injected mice it was evident that the emission in the front paws was higher than in hind paws. For this reason, in Table 1, UPE intensity of the front and hind paws (for the control and experimental group) are described separately before luminol was injected as well as after luminol was injected.

**Table 1:** Average intensities values and standard deviation of the 5 ROI’s on front and hind paws for control and CII animals before and immediately after the injection of luminol.

	Before luminol			After luminol		
	Control animals	CII animals	p	Control animals	CII animals	p
Front	46.4±9.1	63.4±14.0	0.005	81.6±14.7	160.1±61.1	0.001
Hind	33.3±6.1	37.5±6.8	0.162	48.6±7.3	103.6±22.8	0.000
Total	39.8±10.1	50.4±17.1	0.022	65.1±20.4	131.9±53.4	0.000

Before luminol injection, the CII-injected mice generally demonstrated increased emission intensities compared with control mice. The measured values before luminol injection documented a significantly higher intensity (p = 0.005) of the front paws of experimental animals compared to the control animals. The hind paws had a smaller intensity than the front paws. The difference between the hind paws of CII injected animals and controls was smaller and not significant. The photon emission intensities of all paws of the animal (i.e., the total sum of the emissions of both left and right, hind and front paws and then averaged) illustrate a significant higher UPE intensity for the experimental than for control animals before luminol injection (p = 0.022).

Table 1, also illustrates the photon emission intensities of the paws of the CII and control group after luminol injection. The intensity of the CII group illustrates a significantly higher UPE intensity than the control group for the front paws (p = 0.001), hind paws (p = 0,000) and the average of all paws (p = 0.000).

Finally, Table 1 illustrates that luminol increased UPE by a factor of roughly 2. The increase by luminol injection was significant for control animals (hind paws p = 0.001; front paws p = 0.000) and experimental animals (hind paws p = 0.000; front paws p = 0.000).

The data suggest that the increase in UPE intensity as a result of the induced arthritis can be significantly observed with, as well as without luminol.

### Correlation between Left and Right

Because UPE was imaged in the early phase of RA development, it was of interest to determine the degree of symmetry in distribution of UPE intensity within an animal. For that reason we focused on the correlation between intensity values left and right. Intensity values of left and right (hind and front paws) were compared for control and CII injected animals before and after luminol injection.

**Figure 3:**
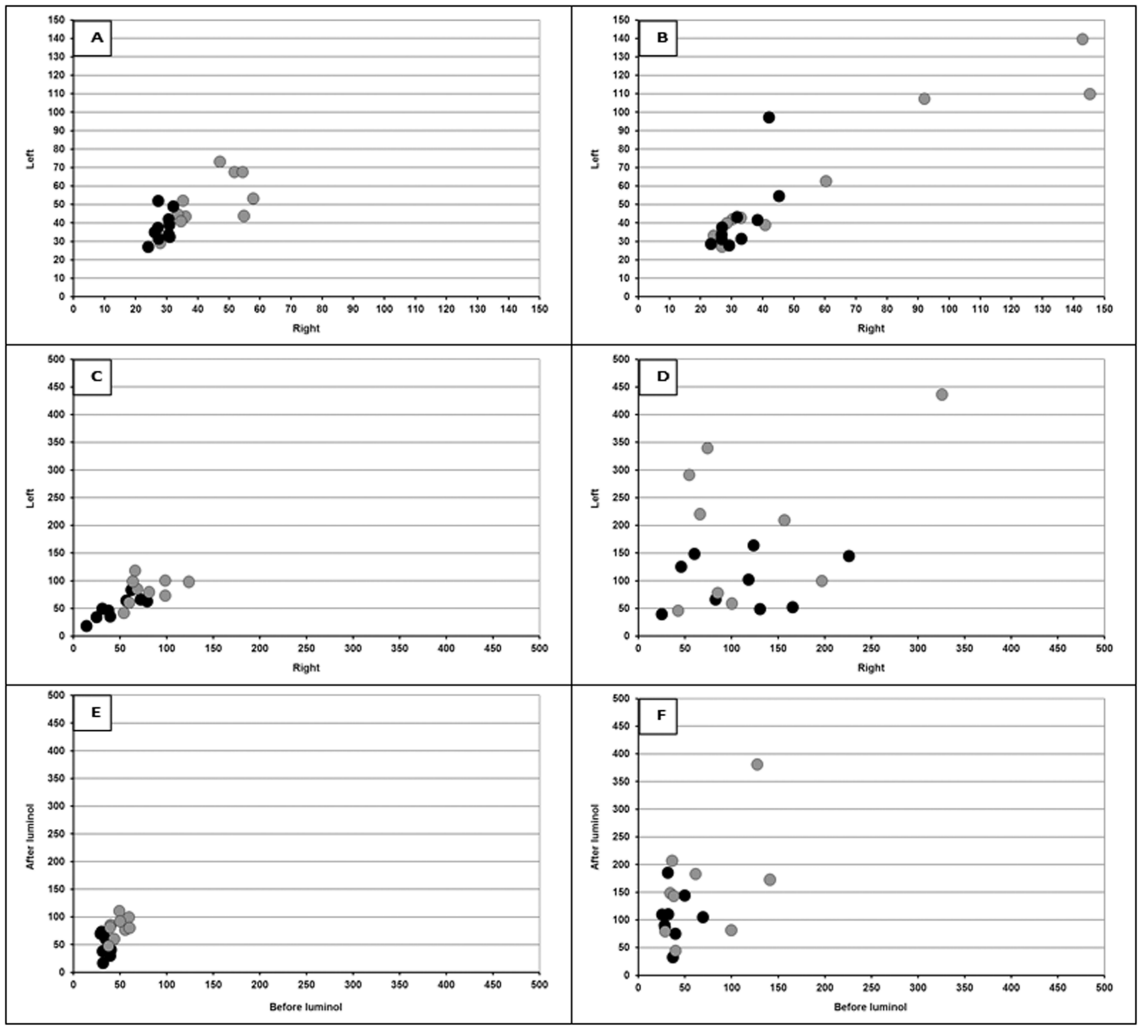
The relationship of UPE intensity between left and right paws. The upper panel presents the left and right intensity values of hind (black •) and front (gray •) paws before luminol injection for the control (Figure 3A) and CII-injected animals (Figure 3B). The middle row of panels of Figure 3 illustrates left and right symmetry in intensity of hind (black circles) and front (grey circles) paws after luminol injection for the control (Figure 3C) and CII injected animals (Figure 3D). The lower panels compares baseline UPE intensity (before luminol) of individual animals with the corresponding increased UPE value after luminol injection of the same animals. The relationship of UPE intensity within an animal (before and after luminol injection) for hind (black circles) and front (grey circles) paws is depicted for the control group (Figure 3E) and CII-injected group (Figure 3F).

Figure 3 illustrates different types of comparisons. The upper panel presents the left and right intensity values of hind and front paws before luminol injection for the control (Figure 3A) and CII-injected animals (Figure 3B). The diagram in Figure 3A illustrates the variance in intensity of control animals ranging from 24–73. In the experimental group, the values range from 23–145 (Figure 3B). The broad range is due to the large difference between the UPE intensities of the front and the hind paws. Therefore, in each panel the hind and front paws are marked so that they can be recognized. In the control animals before luminol, the intensities are relatively low, but small differences between individual animals have been estimated for both front and hind paws. In the experimental group (Figure 3B) the values of front and hind paws are more separated than in the control group (Figure 3A). In both groups, the correlation between left and right paw intensity was estimated, both for front and for hind paws. In the control animals the left-right correlation for the front paws is 0.6434 (p = 0.045) and for the hind paws 0.4029 (n.s.). In the experimental animals, the correlation for the front paws is 0.9609 (p = 000) and for the hind paws 0.7179 (p = 0.019).

The middle row of panels of Figure 3 illustrates left and right symmetry in intensity after luminol injection. The intensity between the animals in the control group ranges from 15–124 (Figure 3C). The intensity in the experimental group varies between 25–436 (Figure 3D). Also, in this case, the left-right correlation was calculated for front and hind paws separately. In general, the left-right correlation after luminol was less and only significant in the hind paws of control animals (r = 0.8467; p = 0.004).

The lower panels focus on the effect of luminol on UPE intensity. It compares baseline UPE intensity (before luminol) of individual animals with the corresponding increased UPE value after luminol injection of the same animals. The relationship of UPE intensity within an animal (before and after luminol injection) is depicted for the control group (Figure 3E) and CII-injected group (Figure 3F). No significant correlation between intensities measured before and after luminol were found, neither for the control group nor for the experimental group.

## Discussion

In summary, data illustrate a higher UPE intensity at 70 days after initiating arthritis by CII- injection of the animals according to the procedure described [28]. The data confirm the results demonstrating the increase in UPE in CII-injected animals after the injection of luminol [24]. The present paper shows that an increase in UPE in CII-injected animals can also be demonstrated before using luminol. This indicates that it is feasible to image increased ROS activity in mice in an early phase of arthritis development without using luminol as an enhancer.

The present protocol for estimating UPE allows a comparison of the same animals before and after luminol injection. An interesting observation is the high variance in UPE intensity between the animals especially in the animals injected with CII. This high variance indicates that in some CII-injected animals arthritis is developed further than in others. The reason is probably because images were made in the early phase of RA development.

Another interesting observation is the correlation between left and right paw intensity in the experimental CII animals before luminol injection (front paws: r = 0.9609; p = 000 and hind paws: r = 0.7179; p = 0.019). In luminol injected animals the left-right correlation was less (front paws: r = 0.4649; p = n.s. and hind paws r = 0.1765; p = n.s.).

The loss of correlation in relationship with the use of luminol regarding UPE intensity was also observed when UPE before luminol injection was compared with the corresponding UPE value after luminol injection. The loss of correlation suggests that luminol diffusion in tissues is highly variable. Thus, the contribution of luminol in order to detect RA documents an opposite trend. On one hand, ROS in the presence of luminol produces more signal. However, on the other hand, the signal has more variation than without luminol. Such may be explained by the reactivity and distribution of luminol throughout the body. This needs to be studied and in case this is true, data without luminol are more reliable (but with less sensitivity).

We envision that the above described technology may be useful for the future study of human RA. Recently major steps have been made in the imaging of human ultra-weak photon emission. With the use of an extremely sensitive CCD camera and lens systems it is possible to image photon emission from larger human body surfaces [30]–[33]. In 2009, the diurnal change of this ultra-weak photon emission measured from large body surfaces was demonstrated [34]. Recently, two-dimensional photon imaging has served as a potential tool for monitoring oxidative stress of human skin induced by various stress factors [35], [36]. Such examples emphasize the potential applications of UPE imaging studies within biology, medicine and environmental studies. It seems likely that this adjunct might rapidly expand *in vivo* imaging repertoire. It might be particularly suited for following ROS levels in real-time and in testing the efficacy of novel therapies.
